# From recognition to action: A strategic approach to foster sustainable collaborations for rabies elimination

**DOI:** 10.1371/journal.pntd.0006756

**Published:** 2018-10-25

**Authors:** Rany Octaria, Stephanie J. Salyer, Jesse Blanton, Emily G. Pieracci, Peninah Munyua, Max Millien, Louis Nel, Ryan M. Wallace

**Affiliations:** 1 Vanderbilt University School of Medicine, Nashville, Tennessee, United States of America; 2 Epidemiology, Informatics, Surveillance, and Laboratory Branch, Division of Global Health Protection, Center for Global Health, Centers for Disease Control and Prevention, Atlanta, Georgia, United States of America; 3 One Health Office, National Center for Emerging and Zoonotic Infectious Disease, Centers for Disease Control and Prevention, Atlanta, Georgia, United States of America; 4 Poxvirus and Rabies Branch, Division of High-Consequence Pathogens and Pathology, National Center for Emerging and Zoonotic Infectious Disease, Centers for Disease Control and Prevention, Atlanta, Georgia, United States of America; 5 Global Disease Detection Center–Kenya, Division of Global Health Protection, Center for Global Health, Centers for Disease Control and Prevention, Nairobi, Kenya; 6 Ministry of Agriculture, Natural Resources and Rural Development, Port-au-Prince, Haiti; 7 Department of Biochemistry, Genetics and Microbiology, University of Pretoria, South Africa and Global Alliance for Rabies Control, Manhattan, Kansas, United States of America; Wistar Institute, UNITED STATES

## Introduction

The World Health Organization (WHO), World Organisation for Animal Health (OIE), Food and Agricultural Organization of the United Nations (FAO), and Global Alliance for Rabies Control (GARC) have established a global goal for the elimination of dog-mediated human rabies deaths by 2030 [1,2]. A significant number of rabies endemic countries have also committed themselves, individually or as a group, to eliminate rabies from their territories. Although tools to eliminate canine rabies are available, financial resources for rabies control are scarce. [2]. Public—private partnerships have shown effective results in the control of certain neglected tropical diseases [3]—like filariasis elimination championed by the Global Alliance for the Elimination of Lymphatic Filariasis (GAELF) [4] and Guinea worm control spearheaded by the Carter Center [5]—and could prove a possible strategy for rabies. The funding to achieve global elimination of dog-mediated human rabies deaths has not yet been realized, and it is unlikely that a single external partner would be able to provide all resources necessary to develop an endemic country’s comprehensive, multiyear rabies control program [5]. Instead, the fiscal investment and infrastructural development will, in many instances, need to be driven in part from the endemic country’s government [2,3]. Indeed, just as rabies elimination is a global public good, national governments should recognize that freedom of dog rabies is a national public good, for which public funds should be invested. National governments should also take the lead in making the final decisions on the overall strategy and the day to day implementation of rabies elimination or control activities. Support from external sources, including international agencies, public entities, donor governments, and private partners, may assist with bridging the funding gap and should aim to fund objectives that align with, or promote, the development of a sustainable government-operated rabies program.

## Existing rabies control resources for countries

Government’s commitment to disease interventions begins with a multisectoral, One Health understanding of the disease burden and agreement that a given disease is a national priority. To facilitate prioritization of a county’s top endemic and emerging zoonotic diseases, the Centers for Disease Control and Prevention (CDC) has developed a One Health Zoonotic Disease Prioritization tool (OHZDP) [6], offered as a two-day facilitated workshop with stakeholders from the human, animal, environmental health, and other relevant sectors. As of May 2018, 19 of 20 countries that have held OHZDP workshops have prioritized rabies [7–14].

For countries that have not prioritized control of rabies or committed to rabies elimination as a national priority, advocacy and increased recognition of the rabies burden may help foster intragovernment support. Several tools for rabies advocacy have been developed in recent years. In 2017, United Against Rabies (UAR), a collaboration of four partners—WHO, the FAO, the OIE and the GARC—was created to advocate for rabies control. UAR fosters a multistakeholder platform to engage countries and experts to advance towards zero dog-mediated human rabies deaths by 2030 [15] (http://www.who.int/rabies/news/RUA-Rabies-launch-plan-achieve-zero-rabies-human-deaths-2030/en/). In some countries intragovernmental support has been fostered through rabies champions—persons who undertake advocacy and awareness roles to motivate communities and governments to action. To support development of rabies champions, the GARC provides annual awards for community leaders [16] and hosts online rabies educational courses [17]. An even more comprehensive educational platform was created by Pasteur Institute, the Customized Online Training (COLT) for rabies control officers [18]. This one-year intensive course trains approximately 30 people per year from numerous countries to be rabies control advocates and experts within their country. The course is hosted online but also includes a two-week on-site workshop and continued mentoring by workshop organizers after the one-year program is completed. Intragovernment support for rabies control has also been fostered through bilateral country agreements targeting regional rabies control and elimination goals. Perhaps the greatest accomplishments in regional rabies control in the past two decades was seen in Latin America, where regional rabies program directors made agreements to commit to canine rabies elimination [19]. This model has been replicated in several regions in just the past several years, with the formation of the ASEAN Rabies Elimination Strategy in 2015 (Brunei, Cambodia, Indonesia, Lao PDR, Malaysia, Myanmar, Philippines, Singapore, Thailand, Viet Nam). In Africa, the Pan-African Rabies Control Network (PARACON) was established in 2015 as the regional network for sub-Saharan African countries [20]. Subsequently, smaller, community-based, subregional planning structures included the Eastern Africa rabies control planning commission and the first meeting of the East Africa Rabies Network in 2017 (Kenya, Tanzania, Rwanda, Ethiopia, Uganda) [21].

In the early stages of rabies control advocacy there are often limited data on the burden of the disease, and data that are available are based on rudimentary surveillance systems that capture only a fraction of human rabies cases [22]. Several burden estimation methods are available to help describe a country’s rabies burden, even where limited data are available. In 2015, the global burden of rabies was estimated by a group of international rabies experts, and country-specific results were published in a supplemental file within the publication [23]. Subsequently, a District Health Information System 2 (DHIS2)-based Rabies Epidemiological Bulletin was developed in order to improve rabies surveillance and the reporting of rabies epidemiological data from Africa; this platform was most recently extended to include the Asian Rabies Control Network (https://rabiesalliance.org/networks/aracon) [20]. Two interactive rabies-burden models are also available. The first is the BioEcon model, developed by the United States Department of Agriculture. This is an individual-based stochastic simulation model to forecast the burden of rabies and economic cost associated with user-defined control measures [24]. The second is a deterministic compartmental model that also estimates the human rabies burden under user-defined scenarios, as well as the economic cost to control the disease through dog vaccination, referred to as the RabiesEcon model [25].

Once countries have committed to rabies control as a priority, it is important to evaluate the current capacity and gaps in logistical capacity, personnel, and financial support. To evaluate a country’s rabies program, the GARC, the FAO, the OIE, and WHO developed the Stepwise Approach towards Rabies Elimination (SARE) tool [26]. During a multiday workshop, stakeholders from the human, animal, and environmental sectors are guided by this Excel-based tool (Microsoft, Redmond, WA) to evaluate their rabies control program and develop a list of high-priority activities to advance their elimination program [26,27]. The SARE tool also links to several international guidance documents, including the WHO Technical Report Series (TRS) on rabies [28], the OIE Terrestrial Manual [29], and the Blueprint for Canine Rabies Control [26].

The Global Dog Rabies Elimination Pathway (GDREP) tool was developed to help estimate the financial and human capital required to achieve canine rabies elimination [2]. This tool describes a three-step approach towards achieving a successful dog rabies vaccination program: preparation, scale-up, and sustainability. The tool is available in an interactive, online format (https://garc.shinyapps.io/gdrep/) [2,30].

These resources provide practical guidance for developing rabies control programs, detailed protocols to conduct field operations, and estimates of the cost, time, and personnel to achieve the goal of rabies elimination. Lacking from these available resources is guidance for how governments and their external partners can foster host government leadership of these rabies control activities.

## Combining resources for sustained control efforts

**Fig 1:**
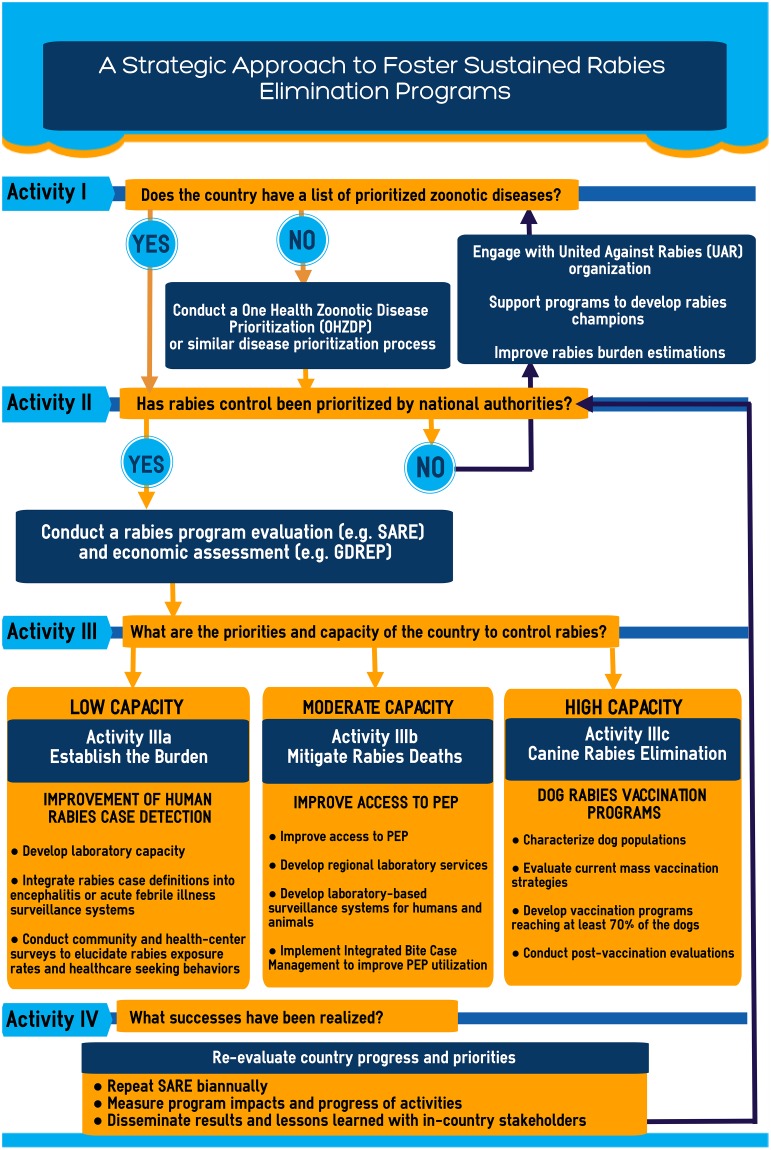
A strategic approach to foster sustained rabies elimination collaborations. GDREP, Global Dog Rabies Elimination Pathway; OHZDP, One Health Zoonotic Disease Prioritization; PEP, post-exposure prophylaxis; SARE, Stepwise Approach for Rabies Elimination.

A proposed strategic approach for coordinating rabies engagement and elimination collaborations (Fig 1) was developed to guide the engagement of government and external partners on canine rabies elimination in a way that promotes host nation leadership. This approach considers that the strengths and utility of current published tools—including OHZDP [6,31], the SARE [26], and the GDREP [2]—are complimentary and can be used as a strategic framework or as individual aids to develop long-lasting government-sponsored rabies control capacity in endemic countries. Desired outcomes, suggested activities, and available resources related to this strategic approach are listed in Table 1.

**Table 1:** Available resources for suggested activities in engagement and elimination of canine rabies in endemic countries.

Activities	Outcomes	Suggested Activities	Available Resources
**I**	**Determination of rabies control as a priority of the host government**	Conduct an OHZDP workshop or similar disease prioritization process that uses a multisectoral, One Health approach	[6,31]
	**Improved intragovernment awareness and advocacy for rabies elimination**	Collaborate with UAR to improve in-country advocacy for rabies control	[15]
		Promote rabies champion development	[16–18]
		Improve rabies case detection	[32]
		Estimate the burden of rabies in the country	[2,23,25]
**II**	**Identification of critical activities necessary to improve the rabies elimination program**	Conduct the SARE workshop or multisectoral evaluation of in-country rabies situation and elimination readiness	[26]
**III a**	**Improve detection of human rabies cases**	Conduct community KAP survey to determine bite rates, healthcare-seeking behavior, human deaths	[33]
		Conduct prospective human rabies investigations using medical center records	[34]
		Establish routine case investigation protocols with verbal autopsy tools	[35]
		Integrate human rabies into existing acute neurologic (encephalitis) or AFI syndromic surveillance system	[36], [37]
		Establish laboratory capacity for detection of human rabies cases	[2,38–40]
**III b**	**Development of an IBCM system to reduce unnecessary PEP usage and decrease human deaths**	Evaluate current surveillance capacity for detection of human and animal rabies cases	[26]
		Establish IBCM protocols	[32]
		Establish PEP treatment recommendations	[26]
		Implement pilot IBCM programs	[32]
		Evaluate impact on healthcare-seeking behaviors	
		Evaluate impact on health economics	[41] [42]
**III c**	**Improvements of canine rabies vaccination coverage to 70% and maintain coverage for five years**	Estimate the cost to implement effective mass dog vaccination programs	[2]
		Describe the dog population	[43,44]
		Evaluate the current vaccination program	[26]
		Develop and implement the ideal vaccination campaign including securing supply of vaccines for the required vaccination coverage and program duration	[45], [46], [26]

**Abbreviations**: AFI, acute febrile illness; CDC, Centers for Disease Control and Prevention; GDREP, Global Dog Rabies Elimination Pathway; IBCM, integrated bite case management; KAP, Knowledge, Attitude, and Practices; MMWR, Morbidity and Mortality Weekly Report; OHZDP, One Health Zoonotic Disease Prioritization; OIE, World Organisation for Animal Health; PEP, post exposure prophylaxis; SARE, Stepwise Approach for Rabies Elimination; UAR, United Against Rabies; WHO, World Health Organization.

### Prioritization (Activity I of Fig 1)

Prioritization of diseases has been performed in a multitude of ways [6,9,47–49] and is a critical first step in gaining government support for building public health capacity. The CDC’s OHZDP tool is one of the few prioritization tools that uses qualitative, quantitative, and semiquantitative methods within a One Health framework by engaging stakeholders from the human, animal, and environmental health sectors as well as other relevant government sectors and their external partners [6,31]. Prioritization through stakeholder engagement is often necessary for diseases like rabies, which require sustained support and collaboration from multiple governmental sectors. When rabies has not been considered a national priority, activities that raise awareness of disease burden and improve in-country advocacy for rabies control should also be pursued.

### Evaluation (Activity II of Fig 1)

Once the efforts to garner the commitment of the country to prioritize rabies control or elimination are successful, conducting a SARE evaluation is a strategic next step to identify critical gaps that may be hindering rabies control progress. The host government and partners should determine the most impactful activities that pertain to short-term rabies control success by considering the outcome of the SARE report [26]. Furthermore, partners and host governments will likely derive considerable benefit from performing an economic landscape analysis (e.g., GDREP) to promote an understanding of the financial goals and timeframe required to achieve disease elimination.

### Strategies for rabies control activities based on country priorities (Activity III of Fig 1)

Approaches to generating comprehensive rabies control strategies were separated into three broad categories based on host government priorities, funding availability, and logistical capacity. Countries with limited logistical capacity and funding for rabies control should consider investing in programs that improve human rabies case detection, in an effort to bolster support for rabies control activities. Countries with moderate logistical and funding capacity should focus on activities that can quickly reduce human rabies deaths. Such activities include improving access to rabies post-exposure prophylaxis (PEP) and improved rabies surveillance systems. Countries with high levels of logistical and fiscal capacity should consider supporting activities that will permanently eliminate canine rabies (e.g., mass dog vaccination). Given the One Health approach required to control rabies, activities will often overlap, and the order in which activities are implemented is flexible and based upon country-specific context.

### Establish the rabies burden (Activity IIIa of Fig 1)

Sustained commitment to rabies control often requires recognition of the disease burden. Burden is best characterized through routine public health surveillance activities to characterize the number of human and animal cases [23]. Laboratory-based surveillance systems can provide quantitative epidemiologic data that can be useful for advocacy and evaluating interventions. WHO, OIE, and the CDC have produced laboratory manuals to guide countries in building laboratory infrastructure [29,50–52].

Countries that lack a dedicated rabies surveillance system may consider integrating human rabies surveillance into an existing program that detects common clinical signs of rabies such as fever or encephalitis. Acute neurologic (encephalitis) or acute febrile illness (AFI) syndromic surveillance programs may provide such a platform for improved case detection [37,53]. Utilization of the WHO case definitions for clinical diagnosis of rabies in humans can provide an indicator of the potential burden without requiring laboratory capacity or a dedicated surveillance program [28].

If the country does not currently have surveillance capacity, community surveys or health center trace-back surveys to elucidate rates of dog bites, human rabies deaths, and health-seeking behaviors can produce valuable information for conducting country-specific burden estimations [54].

### Mitigate human rabies deaths (Activity IIIb of Fig 1)

Human rabies deaths can be drastically reduced through improved access to vaccines for persons with suspected rabies exposures [1]. Provision of rabies PEP may not be a sustainable solution to eliminate human rabies deaths, as demand can quickly overwhelm resources if the virus is not controlled in the reservoir population. More judicious use of PEP can be achieved by integrating surveillance activities and diagnostic findings with PEP decision algorithms. Development of regional laboratory services is also a possible means of reducing treatment delays and the provision of unnecessary treatments, especially in remote areas. Integrated bite case management (IBCM) programs can improve the delivery of PEP to persons with high-risk exposures while simultaneously reducing the frequency in which nonrabies exposures lead to unnecessary vaccination [32,42].

For various reasons, countries often opt to initiate IBCM through pilot projects [55]. Pilot projects allow the development and evaluation of tailored programs, which consider the unique cultural, logistical, and financial situations among the many diverse rabies-endemic countries. Pilot projects also allow countries to showcase an intervention’s feasibility, utility, and cost effectiveness, which would justify national expansion of the program [33,56].

### Rabies elimination through mass dog vaccination (Activity IIIc of Fig 1)

Countries that currently have the desire and resources to eliminate canine rabies and have implemented programs to increase access to vaccines for persons with rabies exposures should focus efforts on mass dog vaccination. These activities should include enumeration of the dog population, evaluation of an array of vaccination strategies, and development of a vaccination program with a goal of ≥70% coverage in endemic areas. Organizations such as World Animal Protection and OIE have developed resources to help planning and evaluation of dog vaccination campaigns, as well as management of stray dog populations; these resources can be found on the rabies control blueprint, section “Case Studies and Documents”.

### Reevaluate country progress and priorities (Activity IV of Fig 1)

The strategic approach considers changing capacity and priorities of the host government to control rabies. The activities proposed by this approach can serve as a general guide rather than a fixed path towards rabies elimination. For example, a country may choose to conduct dog vaccination programs, an activity in Activity IIIc, while building a pilot IBCM project, an activity in Activity IIIb. The approach incorporates frequent reevaluation of country progress and priorities by repeating the SARE evaluation every two years. The government and their external partners can also reevaluate their progress and priorities by frequent measurement of program impacts and progress of activities. Results and lessons learned should be shared with all partners to improve program advocacy.

## Models of success

### Kenya

Much of Kenya’s progress in rabies elimination has been noted by the activities conducted between 2000 and 2015, including their initial efforts to describe the epidemiology of rabies and assess the most effective vaccination strategy for rabies elimination through empirical data and mathematical modeling [57,58]. In 2012, the Kenyan government established the Kenya Zoonotic Disease Unit (ZDU) [59] to improve communication between the Ministry of Health and Ministry of Agriculture Livestock and Fisheries, with the mandate of fostering collaboration between key health sectors for the management of zoonotic diseases. One of the primary goals of the ZDU was to enhance or build zoonotic epidemic and endemic disease surveillance and to coordinate implementation of control measures. As part of the development of this strategic plan, rabies was prioritized as one of the zoonotic diseases targeted for enhanced surveillance and control (Activities I and II). Rabies was again prioritized during a formal OHZDP workshop in 2015 (Activities I and II) [9].

The establishment of the ZDU has been a catalyst for the rabies control program in Kenya and the region. Between 2012 and 2014, the ZDU organized multistakeholder forums for rabies experts in the country to develop a rabies elimination plan. The plan gained further support and was strengthened in 2015, when the country conducted a SARE workshop. The GDREP was conducted in 2017 (Activity II). This elimination plan has attracted donor support for canine rabies vaccination and surveillance campaigns in two pilot counties, which will inform implementation in other regions (Activities IIIb and IIIc) [60]. In addition, existing community-based AFI surveillance has incorporated a specific syndromic component to capture animal health information, which is expected to provide more information about community-based rabies cases in animals (Activity IIIa). Kenya also recognizes that rabies is a transboundary disease, therefore the ZDU—with support from the CDC and the GARC—held the first Eastern Africa regional meeting on rabies control in 2017 to strengthen support and collaboration for regional rabies elimination [21].

### Haiti

At the time of initial engagement in 2011, Haiti’s surveillance system detected, on average, 10 annual human rabies deaths, while the international community and RabiesEcon modelling estimated more than 100 deaths [23,25,32]. This discrepancy influenced the government to place rabies within the scope of their priorities. Suitable zoonotic disease prioritization tools [6] were not available at the start of this project, so the CDC and host government partners focused initial efforts on developing country-specific burden estimates and improving animal and human rabies surveillance (Activities I and II) [33,56]. Revised rabies burden estimates (133 annual deaths) were calculated from the RabiesEcon model using Haitian-derived data and were reported to the Haitian government in 2014 (Activity I) [25,32]. Additionally, the CDC assisted in the development of a rabies laboratory to improve human and animal case detection (Activities IIIa and IIIb).

In 2013, Haiti’s national rabies laboratory was established, and a passive animal rabies surveillance system was implemented with combined inputs from the agriculture and public health sectors. This activity served to increase the awareness of rabies (an 18-fold increase in detection of rabid dogs was demonstrated within the first 6 months of operation), and it functions as a platform for human and animal health workers to collaboratively investigate human and animal rabies cases (Activities IIIa and IIIb) [32].

Engagement between Haitian government and several external partner agencies grew between 2014 and 2017. A formal SARE workshop was conducted in 2015 and GDREP in 2016 (Activity II), and the national rabies control plan was updated to reflect priority activities. Several educational trainings were supported by the CDC and the GARC, which included a workshop in which over 40 Haitians were awarded the Rabies Educator Certificate (Activity I) [17]. The Ministry of Agriculture used data collected through surveys and surveillance activities to successfully apply for World Bank funding to establish a national animal rabies surveillance program in 2016 and scale-up national canine rabies vaccination coverage (>200,000 dogs vaccinated in 2017) (Activities IIIc and IV).

At the onset of this collaboration in 2011, the Haitian government and their external partners had conflicting opinions as to the priority of rabies control among the numerous public health issues impacting Haiti. Therefore, initial efforts were directed towards obtaining government commitment and support by demonstrating the disease burden [32,56]. Haiti is a positive example of how a collaborative rabies control program does not always need to be approached in a stepwise fashion; rather the tools and approaches described above can be implemented strategically, throughout the evolution of the program.

## Conclusion

Rabies elimination requires a One Health approach with long-term and large-scale commitments, both from international entities as well as host governments. In many situations, external partners of the governments of rabies-endemic countries will likely be needed to provide technical expertise or to supplement fiscal and logistical gaps. However, sustainable rabies elimination programs are based on host government leadership [2,3]. Disease prioritization is an important first step for governments as well as partner organizations to ensure that expectations are aligned with a common goal. The tools to assist information gathering, planning, and collaborations from both human and animal health sectors are publicly available. These tools can be applied sequentially or strategically and iteratively during the multiyear progression towards canine rabies elimination.

## References

[1] World Health Organization. Global framework to eliminate human rabies transmitted by dogs by 2030 [Internet]. World Health Organization; 2016. http://www.who.int/neglected_diseases/news/Global_framework_eliminate_human_rabies_transmitted_dogs_2030/en/. [cited 2017 Oct 13].

[2] Wallace RM, Undurraga EA, Blanton JD, Cleaton J, Franka R. Elimination of Dog-Mediated Human Rabies Deaths by 2030: Needs Assessment and Alternatives for Progress Based on Dog Vaccination. Front Vet Sci. 2017; 10.3389/fvets.2017.00009 28239608 PMC5300989

[3] Taylor L, Partners for Rabies Prevention. Eliminating canine rabies: The role of public—private partnerships. Antiviral Res. 2013;98: 314–318. 10.1016/j.antiviral.2013.03.002 23499647

[4] Ottesen EA. Editorial: The Global Programme to Eliminate Lymphatic Filariasis. Trop Med Int Heal. Blackwell Science Ltd; 2000;5: 591–594. 10.1046/j.1365-3156.2000.00620.x 11044272

[5] Dunn C, Callahan K, Katabarwa M, Richards F, Hopkins D, Withers PC, et al The Contributions of Onchocerciasis Control and Elimination Programs toward the Achievement of the Millennium Development Goals. PLoS Negl Trop Dis. 2015 10.1371/journal.pntd.0003703 25996946 PMC4440802

[6] Rist CL, Arriola CS, Rubin C. Prioritizing zoonoses: A proposed one health tool for collaborative decision-making. PLoS ONE. 2014; 10.1371/journal.pone.0109986 25302612 PMC4193859

[7] Salyer SJ, Silver R, Simone K, Behravesh CB. Prioritizing Zoonoses for Global Health Capacity Building—Themes from One Health Zoonotic Disease Workshops in 7 Countries, 2014–2016. Emerg Infect Dis J. 2017;23 10.3201/eid2313.170418 29155664 PMC5711306

[8] Barton C. One Health Zoonotic Disease Prioritization. In: Centers for Disease Control and Prevention [Internet]. 2018. http://preparednessandresponse.org/wp-content/uploads/2018/02/Casey-Barton-Behravesh_PMAC-One-Health-Zoonotic-Disease-Prioritization_28Jan2018-distr.pdf. [cited 2018 May 18].

[9] Munyua P, Bitek A, Osoro E, Pieracci EG, Muema J, Mwatondo A, et al Prioritization of zoonotic diseases in Kenya, 2015. PLoS ONE. 2016;11: 1–11. 10.1371/journal.pone.0161576 27557120 PMC4996421

[10] Sierra Leone Identifies Priority Diseases. In: Preparedness& Response: One Health in Action [Internet]. 2017. http://preparednessandresponse.org/news/sierra-leone-identifies-priority-diseases/. [cited 2018 May 18].

[11] Rwanda One Health Zoonotic Disease Prioritization [Internet]. 2017. http://www.rbc.gov.rw/fileadmin/user_upload/RWANDA_OH_ZOONOTIC_DISEASES_PRIORITIZATION_REPORT.pdf. [cited 2018 October 5].

[12] Kalam A. Bangladesh Prioritizes Diseases. In: Preparedness& Response: One Health in Action [Internet]. 2017. http://preparednessandresponse.org/news/bangladesh-prioritizes-diseases/. [cited 2018 May 18].

[13] Kunateh AM. 2.7 Million People Die of Zoonotic Diseases Annually; FAO, CDC Vow to Reduce the Number -. In: African Eye Report [Internet]. 2018. https://africaneyereport.com/2-7-million-people-die-of-zoonotic-diseases-annually-fao-cdc-vow-to-reduce-the-number/?pr=59301&lang=fr. [cited 2018 May 18].

[14] Centers for Disease Control and Prevention. Zoonotic Disease Prioritization | One Health | CDC [Internet]. https://www.cdc.gov/onehealth/global-activities/prioritization.html. [cited 2018 May 18].

[15] Minghui R., Stone M., Semedo M.H., Nel L., 2018 New global strategic plan to eliminate dog-mediated rabies by 2030. Lancet Glob. Heal. 4–5. 10.1016/S2214-109X(18)30302-4 29929890

[16] Global Alliance for Rabies Control. 2017 World Rabies Day Awards [Internet]. 2017. https://rabiesalliance.org/world-rabies-day/awards. [cited 2018 Jan 19].

[17] Global Alliance for Rabies Control. GARC Education Platform (GEP) [Internet]. https://rabiesalliance.org/capacity-building/gep. [cited 2018 Jan 19].

[18] Bourhy H, Troupin C, Faye O, Meslin F-X, Abela-Ridder B, Sall AA, et al Customized online and onsite training for rabies-control officers. Bull World Health Organ. 2015;93: 503–506. 10.2471/BLT.14.149849 26170509 PMC4490815

[19] Freire de Carvalho M, Vigilato MAN, Pompei JA, Rocha F, Vokaty A, Molina-Flores B, et al Rabies in the Americas: 1998–2014. Rupprecht CE, editor. PLoS Negl Trop Dis. Public Library of Science; 2018;12: e0006271 10.1371/journal.pntd.0006271 29558465 PMC5877887

[20] Scott T.P., Coetzer A., Fahrion A.S., Nel L.H., 2017 Addressing the disconnect between the estimated, reported, and true rabies data: The development of a regional African Rabies Bulletin. Front. Vet. Sci. 4 10.3389/fvets.2017.00018 28265562 PMC5316526

[21] Pieracci EG, Scott TP, Coetzer A, Athman M, Mutembei A, Kidane AH, et al The Formation of the Eastern Africa Rabies Network: A Sub-Regional Approach to Rabies Elimination. Trop Med Infect Dis. Multidisciplinary Digital Publishing Institute; 2017;2: 29 10.3390/tropicalmed2030029 28845466 PMC5568643

[22] Taylor LH, Knopf L. Surveillance of Human Rabies by National Authorities—A Global Survey. Zoonoses Public Health. 2015; 10.1111/zph.12183 25683444

[23] Hampson K, Coudeville L, Lembo T, Sambo M, Kieffer A, Attlan M, et al Estimating the Global Burden of Endemic Canine Rabies. PLoS Negl Trop Dis. 2015; 10.1371/journal.pntd.0003709 25881058 PMC4400070

[24] United States Department of Agriculture. BioEcon [Internet]. https://bioecon.shinyapps.io/CanineRabiesWebApp/. [cited 2018 Jan 23].

[25] Borse R. H., Atkins C. Y., Gambhir M., Undurraga E. A., Blanton J. D., Dyer J. L., Rupprecht C. E., M MI. Cost-effectiveness of dog rabies vaccination programs in East Africa. PLoS Negl Trop Dis. 2018; 10.1371/journal.pntd.0006490 PMC5988334 29791440

[26] OIE, WHO, FAO, The Global Alliance for Rabies Control. The Stepwise Approach towards Rabies Elimination [Internet]. https://caninerabiesblueprint.org/A-stepwise-approach-to-planning. [cited 2018 October 5].

[27] Global Alliance for Rabies Control. Editorial: Strengthening the Stepwise Approach towards Rabies Elimination (SARE) [Internet]. https://rabiesalliance.org/index.php/news/editorial-strengthening-stepwise-approach-towards-rabies-elimination-sare. [cited 2017 Oct 19].

[28] World Health Organization. WHO Technical Report Series 982 Expert Consultation on Rabies: Second Report. World Health Organization; 2013. 24069724

[29] World Organization for Animal health. Rabies (infection with Rabies Virus). Manual of Diagnostics Tests and Vaccines for Terrestrial Animals. 2017. http://www.oie.int/fileadmin/Home/eng/Health_standards/tahm/2.01.17_RABIES.pdf. [cited 2018 October 5].

[30] Wallace R, Undurraga E, Blanton J, Cleaton J, Franka R. Planning aid for the control of dog-mediated human rabies deaths based on dog vaccination [Internet]. https://garc.shinyapps.io/gdrep/. [cited 2018 Jan 23].

[31] Centers for Disease Control and Prevention. One Health: Global Activities [Internet]. http://www.cdc.gov/onehealth/global-activities/index.html. [cited 2018 Oct 5].

[32] Wallace RM, Reses H, Franka R, Dilius P, Fenelon N, Orciari L, et al Establishment of a Canine Rabies Burden in Haiti through the Implementation of a Novel Surveillance Program. PLoS Negl Trop Dis. 2015; 10.1371/journal.pntd.0004245 26600437 PMC4657989

[33] Fenelon N, Dely P, Katz MA, Schaad ND, Dismer A., Moran D, et al Knowledge, attitudes and practices regarding rabies risk in community members and healthcare professionals: Pétionville, Haiti, 2013. Epidemiol Infect. 2017;145: 1624–1634. 10.1017/S0950268816003125 28290915 PMC5426290

[34] Pieracci EG, Schroeder B, Mengistu A, Melaku A, Shiferaw M, Blanton JD, et al *Notes from the Field*: Assessment of Health Facilities for Control of Canine Rabies—Gondar City, Amhara Region, Ethiopia, 2015. MMWR Morb Mortal Wkly Rep. 2016;65: 456–457. doi: 10.15585/mmwr.mm6517a4 27149318 10.15585/mmwr.mm6517a4

[35] Wallace RM, Etheart MD, Doty J, Monroe B, Crowdis K, Dilius Augustin P, et al Dog-mediated human rabies death, Haiti, 2016. Emerg Infect Dis. 2016; 10.3201/eid2211.160826 27767911 PMC5088043

[36] World Health Organization. WHO recommended surveillance standards, Second edition [Internet]. WHO. World Health Organization; 2015. http://www.who.int/csr/resources/publications/surveillance/WHO_CDS_CSR_ISR_99_2_EN/en/. [cited 2018 Oct 5].

[37] Petersen B, Rupprecht C. Human Rabies Epidemiology and Diagnosis. Non-Flavivirus Encephalitis. InTech; 2011 10.5772/21708

[38] Centers for Disease Control and Prevention. Protocol for Postmortem Diagnosis of Rabies in Animals by Direct Fluorescent Antibody Testing [Internet]. https://www.cdc.gov/rabies/pdf/rabiesdfaspv2.pdf. [cited 2018 Jan 23].

[39] Center for Disease Control and Prevention. Biosafety in Microbiological and Biomedical Laboratories [Internet]. pp. 21–1112. https://www.cdc.gov/biosafety/publications/bmbl5/bmbl.pdf. [cited 2018 Jan 23].

[40] World Health Organization. Diagnosis: An overview of laboratory techniques in the diagnosis and prevention of rabies and in rabies research. In: WHO [Internet]. World Health Organization; 2010. http://www.who.int/rabies/human/diagnosis/en/. [cited 2018 Jan 23].

[41] Etheart MD, Kligerman M, Augustin PD, Blanton JD, Monroe B, Fleurinord L, et al Effect of counselling on health-care-seeking behaviours and rabies vaccination adherence after dog bites in Haiti, 2014–15: a retrospective follow-up survey. Lancet Glob Heal. 2017;5: e1017–e1025. 10.1016/S2214-109X(17)30321-2 28911750 PMC5639700

[42] Undurraga EA, Meltzer MI, Tran CH, Atkins CY, Etheart MD, Millien MF, et al Cost-Effectiveness Evaluation of a Novel Integrated Bite Case Management Program for the Control of Human Rabies, Haiti 2014–2015. Am J Trop Med Hyg. The American Society of Tropical Medicine and Hygiene; 2017;96: 1307–1317. 10.4269/ajtmh.16-0785 28719253 PMC5462564

[43] Belo VS, Werneck GL, da Silva ES, Barbosa DS, Struchiner CJ. Population Estimation Methods for Free-Ranging Dogs: A Systematic Review. Kaminski J, editor. PLoS ONE. Public Library of Science; 2015;10: e0144830 10.1371/journal.pone.0144830 26673165 PMC4684217

[44] Global Alliance for Rabies Control. What techniques are available to estimate the number of dogs? [Internet]. 2010. https://caninerabiesblueprint.org/5-4-1-What-techniques-are. [cited 2018 May 25].

[45] Lembo T, Hampson K, Kaare MT, Ernest E, Knobel D, Kazwala RR, et al The feasibility of canine rabies elimination in Africa: Dispelling doubts with data. PLoS Negl Trop Dis. 2010; 10.1371/journal.pntd.0000626 20186330 PMC2826407

[46] Zinsstag J, Durr S, Penny MA, Mindekem R, Roth F, Gonzalez SM, et al Transmission dynamics and economics of rabies control in dogs and humans in an African city. Proc Natl Acad Sci. 2009;106: 14996–15001. 10.1073/pnas.0904740106 19706492 PMC2728111

[47] Trang DT, Siembieda J, Huong NT, Hung P, Ky VD, Bandyopahyay S, et al Prioritization of zoonotic diseases of public health significance in Vietnam. J Infect Dev Ctries. 2015;9: 1315–1322. 10.3855/jidc.6582 26719937

[48] Stebler N, Schuepbach-Regula G, Braam P, Falzon LC. Use of a modified Delphi panel to identify and weight criteria for prioritization of zoonotic diseases in Switzerland. Prev Vet Med. Elsevier B.V.; 2015;121: 165–169. 10.1016/j.prevetmed.2015.05.006 26036342

[49] Pieracci EG, Hall AJ, Gharpure R, Haile A, Walelign E, Deressa A, et al Prioritizing zoonotic diseases in Ethiopia using a one health approach. One Heal. 2016; 10.1016/j.onehlt.2016.09.001 28220151 PMC5315415

[50] World Health Organization. Diagnosis: An overview of laboratory techniques in the diagnosis and prevention of rabies and in rabies research. In: WHO. World Health Organization; 2010.

[51] Centers for Disease Control and Prevention. Protocol for Postmortem Diagnosis of Rabies in Animals by Direct Fluorescent Antibody Testing [Internet]. https://www.cdc.gov/rabies/pdf/rabiesdfaspv2.pdf. [cited 2018 April 30].

[52] Centers for Disease Control and Prevention. Biosafety in Microbiological and Biomedical Laboratories [Internet]. pp. 21–1112. https://www.cdc.gov/labs/pdf/CDC-BiosafetyMicrobiologicalBiomedicalLaboratories-2009-P.PDF. [cited 2018 April 30].

[53] Centers for Disease Control and Prevention. Signs and Symptoms—Rabies [Internet]. https://www.cdc.gov/rabies/symptoms/index.html. [cited 2017 Oct 19].

[54] Fenelon N, Dely P, Katz MA, Schaad ND, Dismer A, Moran D, et al Knowledge, attitudes and practices regarding rabies risk in community members and healthcare professionals: Pétionville, Haiti, 2013. Epidemiol Infect. 2017;145: 1624–1634. 10.1017/S0950268816003125 28290915 PMC5426290

[55] Banyard AC, Horton DL, Freuling C, Müller T, Fooks AR. Control and prevention of canine rabies: The need for building laboratory-based surveillance capacity. Antiviral Research. 2013 10.1016/j.antiviral.2013.04.004 23603498

[56] Schildecker S, Millien M, Blanton JD, Boone J, Emery A, Ludder F, et al Dog Ecology and Barriers to Canine Rabies Control in the Republic of Haiti, 2014–2015. Transbound Emerg Dis. 2017;64: 1433–1442. 10.1111/tbed.12531 27313170

[57] Kagira JM, Kanyari PWN. An Appraisal of Rabies Occurence and Control in Kisumu, Kenya. East Afr Med J. 2012;89: 59–63. http://www.ncbi.nlm.nih.gov/pubmed/26845813. 26845813

[58] Kitala PM, McDermott JJ, Coleman PG, Dye C. Comparison of vaccination strategies for the control of dog rabies in Machakos District, Kenya. Epidemiol Infect. 2002;129: 215–22. http://www.ncbi.nlm.nih.gov/pubmed/12211590 12211590 10.1017/s0950268802006957 PMC2869868

[59] Mbabu M, Njeru I, File S, Osoro E, Kiambi S, Bitek A, et al Establishing a One Health office in Kenya. Pan Afr Med J. 2014;19: 106 25722779 10.11604/pamj.2014.19.106.4588 PMC4337352

[60] Republic of Kenya Zoonotic Disease Unit. National Strategic Plan for the Implementation of One Health Kenya 2012–2017 [Internet]. Nairobi; 2014. http://zdukenya.org/wp-content/uploads/2012/09/ZDU_StrategicPlan_updated.pdf. [cited 2018 March 13].

